# Watermelon Intake Is Associated with Increased Nutrient Intake and Higher Diet Quality in Adults and Children, NHANES 2003–2018

**DOI:** 10.3390/nu14224883

**Published:** 2022-11-18

**Authors:** Kristin Fulgoni, Victor L. Fulgoni

**Affiliations:** Nutrition Impact, LLC, Battle Creek, MI 49014, USA

**Keywords:** NHANES, watermelon, diet quality, nutrient intake, fruit

## Abstract

Watermelon is a nutrient-dense, low energy food that provides vital nutrients and contributes to overall fruit intake. Previous studies have found positive associations between watermelon and nutrient intake but few focused on raw watermelon intake or had small sample sizes. Therefore, the objective of this study was to utilize a large, nationally representative sample to determine associations between watermelon intake and nutrient intake and diet quality. Data from children (2–18 y) and adults (19+ y) who participated in the National Health and Nutrition Examination Survey (NHANES) cycles 2003–2018 were utilized in the current study. Watermelon intake was 7.51 and 7.29 g/d per capita in children and adults, respectively. In watermelon consumers, usual intake was 125 and 161 g/d in children and adults, respectively. Total diet quality was higher in watermelon consumers as compared to non-consumers as well as several subcomponent scores. Children and adult watermelon consumers had greater than 5% higher intake of dietary fiber, magnesium, potassium, and vitamin A as well as more than 5% lower intake of added sugars and total saturated fatty acids as well as higher intake of lycopene and other carotenoids. This study suggests watermelon can increase nutrient intake as well as diet quality in both children and adult Americans.

## 1. Introduction

The Dietary Guidelines for Americans (DGA) recommends 1.5 to 2.5 cups of fruit daily due to its nutrient-dense, low energy status as well as of its association with lower risk of all-cause mortality, cardiovascular disease, overweight and obesity, type 2 diabetes, detrimental bone health, and colorectal and breast cancers [1,2]. Additionally, fruits provide vital nutrients to the diet including potassium, magnesium, vitamins A, C, and E, folate, and fiber while contributing minimally to sodium, added sugars, and saturated fats and may reduce overall energy intake [1]. Currently, both US children and adults fall short of consuming the recommended fruit servings and only consume 1.10 and 0.88 cup equivalents per day, respectively [3]. A higher percentage of younger children consume the recommended amount of fruit, but fruit intake decreases during childhood resulting in lower percentages of older adolescents meeting recommendations [1,3]. In adults, the average fruit intake is below recommended levels with younger adults consuming less servings than their older counterparts [1,3].

Watermelon is a member of the gourd family (*cucurbitaceae*) and falls under the Food patterns equivalents database (FPED) category of citrus, melons, and berries [3]. While in the past watermelon was typically thought of as a seasonal fruit in the US, now watermelon is available year-round [4]. It contributes certain nutrients, with 100 g providing 112 mg of potassium, 8.1 mg of vitamin C, 28 µg of vitamin A, 10 mg of magnesium, 3 µg of folate and 0.4 g of dietary fiber contributing upwards of 4% of daily potassium, 11–37% of vitamin C, and 4–10% of vitamin A as well as greater than 1% of magnesium and dietary fiber recommendations in adults and children [5,6,7,8,9,10,11]. Additionally, watermelon has high bioavailability of antioxidant components including lycopene and l-citrulline [5,12,13]. Studies focused on watermelon supplements and/or extracts have shown benefits on the cardiovascular system including decreased pulse pressure, and systolic and diastolic blood pressures in prehypertensive and hypertensive subjects as well as decreased total and LDL cholesterol in subjects with dyslipidemia [14,15,16]. While positive results have been reported from consuming watermelon supplements and/or extracts, the treatments often equated to large raw watermelon intakes, equating to over 2 pounds in a day. Studies focused on raw watermelon intake are less common but have reported reduced triglycerides and LDL cholesterol, body weight, BMI, lower risk of prostate, lung, and breast cancer, as well as higher antioxidant capacity [17,18,19,20]. These studies are important for our understanding of the benefits of raw watermelon intake but the generalization of results is limited due to small sample sizes.

While the nutrient intake and diet quality benefits of fruits has been well studied, a focused study of the impact of watermelon intake on these variables is lacking. Therefore, the goal of the current study was to assess the differences in nutrient intake and diet quality of watermelon consumers and non-consumers in a large sample size representative of the US population.

## 2. Materials and Methods

### 2.1. Data Source and Subjects

The National Health and Nutrition Examination Survey (NHANES) is a government run continuous survey representative of the non-institutionalized United States population that includes a dietary intake component, What We Eat in America (WWEIA). WWEIA subjects report their intake of food over 1 or 2 24 h periods and is collected using the Automated Multiple Pass Method (AMPM) [21]. WWEIA consists of 15 main food groups, 46 subcategories, and over 150 unique categories and individual food intakes and is used in conjunction with relevant Food Patterns Equivalent Databases [22,23]. Collection methods are standardized and are available on the NHANES website [24,25]. NHANES methods have been approved by the Research Ethic Review Board and identifying data is not publicly available.

The current study utilized NHANES cycles 2003–2004, 2005–2006, 2007–2008, 2009–2010, 2011–2012, 2013–2014, 2015–2016, and 2017–2018 in which all cycles were designed to have two days of dietary recall which is needed for usual intake determination. Data from male and female participants 2 years and older with complete 24 h dietary recalls were analyzed. Pregnant and/or lactating subjects (*n* = 1130) and subjects with incomplete or unreliable dietary reports (*n* = 17,239) were removed from the sample, resulting in a sample size of 56,133. Individuals with the most knowledge of their dietary intake were used as proxies for subjects 12 years old and younger. Watermelon consumers were defined as subjects who reported raw watermelon or 100% watermelon juice food codes (63149010 and 64133100, respectively) during their 24 h recall dietary interview. Intake was analyzed using a single 24 h recall and usual intake (UI), a measure of longer-term intake using the National Cancer Institute method [26]. Day 1 consumers were defined as subjects who reported consuming watermelon on the first 24 h dietary recall. For usual intake, the first and second 24 h dietary recalls were used with the National Cancer Institute method. Given episodic consumption of watermelon, the two-part model (frequency and amount) was used. For demographics, Healthy Eating Index 2015 (HEI-2015), nutrient intake, and trend in intake (supplementary analysis), data for day 1 subjects were utilized. Watermelon intake per capita and by consumers were analyzed for both day 1 and UI. As we were concerned with the possibility that trends in watermelon intake may affect our results, we conducted an analysis of intake over NHANES cycles. Since no significant trends were observed, we combined the data of all NHANES cycles for subsequent analyses (Supplementary Table S1).

### 2.2. Nutrient Intake and Diet Quality Assessment

Diet quality was assessed using the HEI-2015 which determines adherence to DGA recommendations [27,28]. Higher scores are associated with higher intake for subcomponents total vegetables, greens and beans, total fruit, whole fruit, whole grains, dairy, total protein foods, seafood and plant protein, and fatty acid ratio, whereas lower intakes of foods recommended to be limited, sodium, refined grains, saturated fat, and added sugars, result in higher scores. Total HEI-2015 scores are calculated as the summation of component scores. Macro and micro-nutrient intakes were determined using Food and Nutrient Database for Dietary Studies (FNDDS) which provides nutrient values for food and beverages reported in WWEIA [29].

### 2.3. Statistical Analyses

All analyses utilized SAS 9.4 (SAS Institute, Cary, NC, USA) and were adjusted for the complex sample design of NHANES. Regression analyses were used to assess the difference of nutrient intake and diet quality between watermelon consumers and non-consumers using PROC SURVEYREG of SAS. Covariates for nutrient intake included energy (except for energy intake), age, gender, ethnicity, physical activity, poverty income ratio (PIR), smoking status, and alcohol intake. Upon review of initial results, we subsequently decided to run some post hoc analyses to assess whether intake of other food groups might be confounded with watermelon intake. Given changes we saw in intakes of vitamin A, vitamin C, magnesium, and potassium, we chose to run additional analyses with total vegetables, non-watermelon fruit intake, and total dairy intake as additional covariates. These additional analyses were run for both HEI-2015 variables as well as intakes for both age groups.

## 3. Results

### 3.1. Demographics

Day 1 child and adolescent watermelon consumers were younger, more likely to be non-Hispanic White, have a PIR > 1.85, have less than or equal to a High School education, have a vigorous activity level, and to have never been a smoker (Table 1). Child and adolescent watermelon consumers were also less likely to be underweight, non-Hispanic Black, have a PIR < 1.35 or 1.35 ≤ PIR ≤ 1.85, have a sedentary or moderate physical activity level, and be former or current smokers. Day 1 adult watermelon consumers were older, more likely to be female, have a PIR > 1.85, and to have never been a smoker (Table 1). Adult consumers were less likely to have a PIR < 1.35 or 1.35 ≤ PIR ≤ 1.85 and to be a current smoker.

### 3.2. Watermelon Intake

The large majority of watermelon intake was provided by raw watermelon (98%) rather than watermelon juice (2%) (data not shown). For day 1, watermelon intake was 7.51 g per day (g/d) per capita in children and adolescents and 7.29 g/d per capita in adults (Table 2). Usual intake of watermelon intake by children and adolescents was 7.73 g/d per capita and 7.43 g/d per capita in adults (Table 2).

### 3.3. Watermelon Consumer Intake

For day 1 children and adolescent watermelon consumers, average intake was 232 g/d and usual intake was 125 g/d (Table 3). The consumer median intake of day 1 watermelon consumers was 152 g/d whereas the usual intake median was 117 g/d. Intake of watermelon was higher in male children and adolescents as compared to females.

In day 1 adult watermelon consumers, average intake was 286 g/d and usual intake was 161 g/d. The median day 1 intake by adult watermelon consumers was 196 g/d whereas the median usual intake was 141 g/d. Similar to their younger counterparts, adult male watermelon consumers had higher intake of watermelon as compared to females.

### 3.4. Diet Quality

Children and adolescent watermelon consumers had higher HEI-2015 component scores for total fruit [+1.8 points (pts)], whole fruit (+2.4 pts), saturated fat (+0.5 pts), and added sugar (+0.7 pts), as well as a higher HEI-2015 total score (+6.6 pts) compared to non-consumers (Table 4).

Adult watermelon consumers had higher HEI-2015 component scores for total vegetables (+0.3 pts), total fruit (+2.3 pts), whole fruit (+2.5 pts), fatty acid ratio (+0.4 pts), saturated fat (+0.8 pts), and added sugar (+0.6 pts) compared to non-consumers (Table 5). Adult watermelon consumers also had a higher total HEI-2015 score (+7.3 pts) as compared to non-consumers.

### 3.5. Nutrient Intake

Child watermelon consumers had lower intake of added sugars (−2.6 tsp eq), total monounsaturated fatty acids (−0.9 g; MUFA), and total saturated fatty acids (−1.4 g; SFA) intake as compared to non-consumers. Children and adolescent watermelon consumers also had higher intake of dietary fiber (+1.8 g), choline (+23 mg), copper (+0.1 mg), magnesium (+32 mg), potassium (+219 mg), and vitamins A (+88 RE), C (+23 mg), and K (+10 mg), beta-carotene (+1059 µg), beta-cryptoxanthin (+190 µg), lutein + zeaxanthin (+255 µg), and lycopene (+10,338 µg) compared to non-consumers (Table 6).

Adult watermelon consumers had lower intake of added sugars (−2.0 tsp eq), total fat (−4.7 g), total MUFA (−1.7 g), total polyunsaturated fatty acids (−0.8 g; PUFA), total SFA (−1.9 g), and higher intake of carbohydrates (+13 g), dietary fiber (+1.2 g), copper (+0.06 mg), magnesium (+22 mg), potassium (+314 mg), thiamin (+0.1 mg), total sugars (+11 g), vitamins A (+92 RE), and C (+41 mg), beta-carotene (+1545 µg), beta-cryptoxanthin (+262 µg), lutein + zeaxanthin (+373 µg), and lycopene (+13,278 µg) compared to non-consumers and lower vitamin B12 (−0.5 µg) (Table 7).

Post hoc analyses results (with total vegetable intake, non-watermelon fruit intake, and total dairy intake as additional covariates) are presented in Supplementary Table 2, Table 3 and Table 4. In both children and adults, the additional covariates attenuated only slightly the higher total HEI-2015 scores in watermelon consumers (53.7 to 53.2 and 57.6 to 56.4, respectively) with the saturated fat sub-component no longer being significant in children and the fatty acid ratio sub-component no longer being significant in adults. In both children and adults, the additional covariates did not change the statistically higher intakes of vitamin A, vitamin C, magnesium, and potassium in watermelon consumers as compared to non-consumers. Vitamin C intakes were reduced some in both age groups (101 to 97 and 123 to 111 mg/d, respectively) but remained 19–30 mg higher than non-consumers.

## 4. Discussion

The results suggest watermelon is a nutrient-dense food that provides numerous nutrients to the diet and contributes to higher diet quality. These data are similar to previous studies which have shown fruit in general, as well as individual fruits such as mangos and apples, contribute to nutrient intakes [1,2,30,31]. As opposed to most fruits, watermelon also has high levels of lycopene, beta-carotene, beta-cryptoxanthin, and lutein and zeaxanthin. Watermelon has over ten and six times higher beta-carotene and beta-cryptoxanthin content, respectively, than other commonly consumed fruits [5]. Encouraging Americans to consume watermelon could benefit intake of certain nutrients as well as unique components with antioxidant properties.

This study provides further evidence of the positive impact fruit and especially watermelon can have on the diet quality of children and adults. Watermelon intake was associated with not only higher overall diet quality score, but also higher subcomponent scores unrelated to fruit, further suggesting their benefit towards adhering to overall dietary recommendations. Previous studies have shown other nutrient-dense foods provide benefits in diet quality subcomponents unrelated to the studied food group, for example oatmeal, mango, and nuts, but the current study suggests watermelon may be associated with larger increases. For example, watermelon provided the highest percent change in total vegetables (3 and 10% increase in children and adults, respectively), protein (5% increase in children), fatty acid ratio (9% increase in children) and saturated fat (9 and 13% increase in children and adults, respectively) scores compared to mango, nuts, and oatmeal which increased total vegetables by 3 and 7.5% (oatmeal and mango in adults, respectively), protein by 3% (only mango in children), 7–8% increase in fatty acid ratio, and 1–5% increase in saturated fat [30,32,33]. It is interesting that watermelon and other fruit consumption were associated with better HEI-2015 component scores unrelated to fruit indicating the selection of a healthier diet. This was evident in consistently higher scores in both children and adults for saturated fat and added sugars component scores. It’s possible the sweetness of watermelon curbed the desire to have other foods with added sugars. It’s unclear why saturated fat scores were consistently higher (and saturated fat intakes lower), but this may also be related to high added sugars scores (and lower added sugars intake) as many foods with added sugars also have higher saturated fat (i.e., cookies and other bakery items). Further research may be warranted to assess whether watermelon consumption could lower added sugars and saturated fat intake in a clinical trial setting.

The post hoc analyses results (with total vegetable intake, non-watermelon fruit intake, and total dairy intake as additional covariates) further supports, but still does not prove, that at least some of the differences in intakes can be attributed to watermelon intake. That said, it also appears that watermelon consumers also select diets more aligned with current US dietary guidance as supported by higher total HEI-2015 scores in watermelon consumers even after adjusting for intakes of total vegetables, non-watermelon fruit, and total dairy.

Like other fruit, benefits other than those measured as diet quality and nutrient intake are likely associated with watermelon intake but studies are limited. A 2019 study found 2 cups of fresh watermelon daily was associated with lower body weight, BMI, hunger rating, and desire to eat, suggesting watermelon is a healthy snack to help lose weight and curb appetite [18]. Another important feature of fruit including watermelon is their ability to contribute to total fluid intake with one study reporting approximately 12% total water intake was provided by fruit [1,34]. While numerous beverages would provide more hydration, consuming foods with high moisture content like fruits, including watermelon which is 91% water by weight, can help meet hydration needs [5]. Hydration status has been associated with numerous health benefits including cognitive functioning and mood [35,36]. Dehydration tends to be more common in older adults [35] and given that the average age of adult consumers in this study was 52 y, it would be beneficial to encourage more older adults to consume watermelon. Future studies could focus on health benefits of watermelon outside of diet quality and nutrient intake including satiety, cardiovascular, cognition, and mood.

The current study has several strengths including a large sample size representative of the US population and assessment of raw watermelon as compared to watermelon concentrate or powder supplements. Additionally, the post hoc analyses adjusting for intakes of total vegetables, non-watermelon fruit, and total dairy further supports at least some of the changes seen in intakes and HEI-2015 scores were attributable to watermelon intake, however given NHANES is an observational study cause and effect cannot be determined. Additionally, this study has limitations; NHANES utilizes dietary recalls which relies on memory and can be sensitive to misreporting [37]. Proxies were used for subjects less than 12 years of age which may be less informed about food intake outside of the house possibly further impacting misreporting. Although multiple covariates were utilized, further confounding variables may be present. Seasonality of watermelon intake was unable to be assessed given this is not available in the publicly available data due to NHANES confidentiality issues.

## 5. Conclusions

In conclusion, watermelon intake was associated with higher nutrient intake and provided high levels of lycopene, beta-carotene, and beta-cryptoxanthin. Intake of watermelon was also associated with higher diet quality and higher scores for some non-fruit related diet quality components. Consumption of watermelon should be encouraged to help improve US children and adult diet quality and nutrient intake.

## Figures and Tables

**Table 1:** Demographic characteristics of children (2–18 years) and adult (19+ years) watermelon consumers and non-consumers, NHANES 2003–2018.

Demographic Variable	Children (2–18 Years)			Adults (19+ Years)		
	Non-Consumers (*n* = 20,438)	Consumers (*n* = 566)	*p* ^a^	Non-Consumers (*n* = 34,241)	Consumers (*n* = 885)	*p*
Age	10.16 (0.07)	9.09 (0.27)	**0.0002**	47.13 (0.23)	51.93 (0.85)	**<0.0001**
Male (%)	50.66 (0.60)	51.10 (3.13)	0.8855	48.99 (0.37)	39.99 (2.03)	**<0.0001**
**Weight Status (%)**						
Underweight	3.44 (0.26)	1.49 (0.62)	**0.0053**	1.55 (0.10)	1.23 (0.52)	0.5518
Normal weight	63.27 (0.62)	67.38 (3.01)	0.1561	29.02 (0.50)	28.62 (2.17)	0.8598
Overweight	15.72 (0.41)	13.29 (2.26)	0.2778	32.52 (0.49)	36.72 (2.92)	0.1637
Obese	17.57 (0.51)	17.84 (2.42)	0.9112	36.91 (0.56)	33.42 (2.41)	0.1522
**Ethnicity (%)**						
Mexican American	15.22 (1.02)	12.19 (2.24)	0.1450	8.44 (0.62)	6.79 (1.40)	0.1874
Other Hispanic	6.45 (0.51)	6.85 (1.32)	0.7375	5.08 (0.39)	4.35 (0.87)	0.3839
White	55.40 (1.61)	66.36 (3.65)	**0.0021**	68.00 (1.21)	69.84 (2.97)	0.5014
Black	14.36 (0.92)	7.65 (1.60)	**<0.0001**	11.43 (0.67)	10.28 (1.30)	0.3390
Other	8.57 (0.48)	6.95 (1.82)	0.3756	7.04 (0.36)	8.75 (1.38)	0.2216
**Poverty Income Ratio (%, PIR)**						
PIR < 1.35	33.98 (1.10)	24.09 (2.52)	**0.0002**	22.40 (0.66)	17.44 (1.83)	**0.0080**
1.35 ≤ PIR ≤ 1.85	10.80 (0.49)	7.78 (1.46)	**0.0384**	9.94 (0.31)	6.96 (1.13)	**0.0127**
PIR > 1.85	55.22 (1.18)	68.13 (3.31)	**0.0001**	67.66 (0.81)	75.60 (2.12)	**0.0003**
**Education (%)**						
Less than High School	99.04 (0.12)	99.99 (0.01)	**<0.0001**	39.38 (0.81)	36.32 (2.73)	0.2597
Between High School and Bachelor Degree	0.96 (0.12)	0.01 (0.01)	**<0.0001**	31.93 (0.49)	30.56 (2.26)	0.5344
Bachelor Degree or Higher	0 (0)	0 (0)		28.69 (0.83)	33.12 (2.74)	0.0937
**Physical Activity (%)**						
Sedentary	14.19 (0.44)	9.31 (1.60)	**0.0026**	24.11 (0.49)	21.57 (1.86)	0.1590
Moderate	22.69 (0.56)	17.25 (2.53)	**0.0362**	35.98 (0.48)	37.47 (2.15)	0.4965
Vigorous	63.12 (0.65)	73.43 (3.01)	**0.0011**	39.91 (0.60)	40.96 (2.35)	0.6637
**Smoking Status (%)**						
Never	89.27 (0.48)	95.04 (1.19)	**<0.0001**	52.88 (0.55)	60.71 (2.92)	**0.0076**
Former	8.55 (0.41)	4.33 (1.13)	**0.0008**	27.54 (0.43)	28.40 (2.53)	0.7323
Current	1.89 (0.19)	0.54 (0.28)	**0.0001**	19.46 (0.52)	10.87 (1.70)	**<0.0001**

Data source: NHANES 2003–2018; subjects 2 years and older with complete, reliable recall on Day 1 (*n* = 56,133). Values are presented as Mean (standard error). ^a^ *p* value for difference between watermelon consumers and non-consumers. Bolded values are significantly different, *p* < 0.05.

**Table 2:** Intake of watermelon (grams/day) per capita of children (2–18 years) and adults (19+ years), NHANES 2003–2018.

	Watermelon Intake (g)	
Gender	Day 1 a	Usual Intake b
Children (2–18 years)		
All (*n* = 21,004)	7.51 (0.81)	7.73 (0.56)
Male (*n* = 10,541)	8.27 (1.22)	8.24 (0.71)
Female (*n* = 10,463)	6.72 (0.87)	7.22 (0.71)
Adults (19+ years)		
All (*n* = 35,126)	7.29 (0.59)	7.43 (0.47)
Male (*n* = 17,226)	6.96 (0.77)	7.24 (0.63)
Female (*n* = 17,900)	7.61 (0.71)	7.61 (0.47)

Data source: NHANES 2003–2018; subjects 2 years and older with complete, reliable dietary recalls (*n* = 56,133). Data presented as Mean (SE). ^a^ Day 1 consumers defined as subjects who reported watermelon on the first 24 h dietary recall. ^b^ Usual intake determined with the National Cancer Institute method and used the first and second 24 h dietary recall.

**Table 3:** Intake of watermelon (grams/day) by child (2–18 years) and adult (19+ years) consumers only, NHANES 2003–2018.

	**2–18 Years**					
**Watermelon Intake Variable**	**All (*n* = 566)**		**Males (*n* = 264)**		**Females (*n* = 302)**	
	**Day 1 ^a^**	**Usual Intake ^b^**	**Day 1**	**Usual Intake**	**Day 1**	**Usual Intake**
Mean (SE)	232.3 (18.5)	124.6 (3.81)	253.8 (28.5)	132.0 (5.83)	209.8 (17.4)	116.8 (3.59)
Median (SE)	151.5 (10.2)	117.0 (3.82)	152.2 (16.7)	127.7 (5.13)	151.1 (11.2)	110.3 (2.00)
**Watermelon Intake Variable**	**19+ Years**					
	**All (*n* = 885)**		**Males (*n* = 374)**		**Females (*n* = 511)**	
	**Day 1**	**Usual Intake**	**Day 1**	**Usual Intake**	**Day 1**	**Usual Intake**
Mean (SE)	286.5 (15.1)	161.5 (3.85)	333.3 (27.6)	187.6 (8.34)	255.3 (17.0)	144.1 (3.82)
Median (SE)	196.3 (11.8)	141.1 (3.15)	253.5 (21.1)	169.2 (6.79)	169.9 (12.4)	136.2 (3.13)

Data source: NHANES 2003–2018; subjects 2 years and older with complete, reliable dietary recalls (*n* = 56,133). ^a^ Day 1 consumers defined as subjects who reported watermelon on the first 24 h dietary recall. ^b^ Usual intake determined with the National Cancer Institute method and used the first and second 24 h dietary recall.

**Table 4:** Association of watermelon consumption with Healthy Eating Index-2015 total and sub-component scores in children, NHANES 2003–2018, gender combined data.

Healthy Eating Index 2015 Components ^a^	Non-Consumer (*n* = 18,821)	Consumer (*n* = 513)		
	Mean (SE)	Mean (SE)	Beta (SE)	*p* ^b^
Component 1–total vegetables	2.15 (0.02)	2.22 (0.10)	0.07 (0.10)	0.4730
Component 2–greens and beans	0.87 (0.03)	1.04 (0.13)	0.17 (0.13)	0.2097
Component 3–total fruit	2.46 (0.03)	4.29 (0.09)	1.83 (0.09)	**<0.0001**
Component 4–whole fruit	2.17 (0.04)	4.56 (0.05)	2.40 (0.06)	**<0.0001**
Component 5–whole grains	2.31 (0.05)	2.63 (0.18)	0.32 (0.18)	0.0852
Component 6–dairy	6.90 (0.04)	6.63 (0.20)	−0.27 (0.21)	0.1887
Component 7–total protein foods	3.56 (0.02)	3.73 (0.10)	0.17 (0.10)	0.0888
Component 8–seafood and plant protein	1.57 (0.03)	1.90 (0.17)	0.32 (0.17)	0.0626
Component 9–fatty acid ratio	3.83 (0.04)	4.16 (0.28)	0.33 (0.28)	0.2378
Component 10–sodium	4.96 (0.05)	5.14 (0.22)	0.17 (0.23)	0.4454
Component 11–refined grain	5.18 (0.05)	5.05 (0.30)	−0.13 (0.29)	0.6652
Component 12–saturated fat	5.42 (0.05)	5.93 (0.25)	0.52 (0.25)	**0.0403**
Component 13–added sugar	5.68 (0.05)	6.41 (0.18)	0.73 (0.19)	**0.0002**
HEI-2015 total score	47.05 (0.19)	53.69 (0.87)	6.64 (0.87)	**<0.0001**

Data source: NHANES 2003–2018; subjects 2 to 18 years old with complete, reliable dietary recall on Day 1 (*n* = 21,004). ^a^ Values are adjusted for age, gender, ethnicity (Mexican American, other Hispanic, White, Black, Other), poverty income ratio, physical activity level (sedentary, moderate, vigorous). ^b^ *p* value for difference of intake between watermelon consumers and non-consumers; Bolded values are significantly different, *p* < 0.05; SE = standard error.

**Table 5:** Association of watermelon consumption with Healthy Eating Index-2015 total and sub-component score in adults, NHANES 2003–2018, gender combined data.

Healthy Eating Index 2015 Components ^a^	Non-Consumer (*n* = 31,569)	Consumer (*n* = 792)		
	Mean (SE)	Mean (SE)	Beta (SE)	*p* ^b^
Component 1–total vegetables	3.07 (0.02)	3.39 (0.08)	0.32 (0.08)	**0.0001**
Component 2–greens and beans	1.49 (0.02)	1.65 (0.13)	0.17 (0.13)	0.1882
Component 3–total fruit	1.99 (0.02)	4.30 (0.06)	2.31 (0.06)	**<0.0001**
Component 4–whole fruit	1.98 (0.03)	4.52 (0.04)	2.54 (0.05)	**<0.0001**
Component 5–whole grains	2.40 (0.04)	2.43 (0.15)	0.03 (0.16)	0.8491
Component 6–dairy	5.09 (0.04)	4.92 (0.20)	−0.17 (0.20)	0.3994
Component 7–total protein foods	4.21 (0.01)	4.21 (0.05)	−0.01 (0.05)	0.9219
Component 8–seafood and plant protein	2.32 (0.02)	2.18 (0.14)	−0.14 (0.14)	0.3313
Component 9–fatty acid ratio	4.96 (0.04)	5.33 (0.17)	0.38 (0.18)	**0.0322**
Component 10–sodium	4.23 (0.03)	4.52 (0.19)	0.29 (0.19)	0.1393
Component 11–refined grain	6.16 (0.03)	6.41 (0.19)	0.25 (0.20)	0.2038
Component 12–saturated fat	5.87 (0.03)	6.64 (0.19)	0.77 (0.19)	**0.0001**
Component 13–added sugar	6.54 (0.04)	7.10 (0.17)	0.56 (0.17)	**0.0017**
HEI-2015 total score	50.29 (0.18)	57.61 (0.61)	7.31 (0.63)	**<0.0001**

Data source: NHANES 2003–2018; subjects 19 years and older with complete, reliable dietary recall on Day 1 (*n* = 35,126). ^a^ Values are adjusted for age, gender, ethnicity (Mexican American, other Hispanic, White, Black, Other), poverty income ratio, physical activity level (sedentary, moderate, vigorous), smoking status. ^b^ *p* value for difference of intake between watermelon consumers and non-consumers; Bolded values are significantly different, *p* < 0.05; SE = standard error.

**Table 6:** Energy and nutrient intakes in children watermelon consumers (*n* = 513) and non-consumers (*n* = 18,821), NHANES 2003–2018, gender combined data.

Nutrients ^a^	Non-Consumer (*n* = 18,821)	Consumer (*n* = 513)		
	Mean (SE)	Mean (SE)	Beta (SE)	*p* ^b^
Added sugars (tsp eq)	18.90 (0.15)	16.32 (0.65)	−2.58 (0.66)	**0.0002**
Beta-carotene (mcg)	1154 (31.1)	2212 (197)	1059 (200)	**<0.0001**
Beta-cryptoxanthin (mcg)	78.69 (2.73)	269.1 (17.7)	190.4 (17.5)	**<0.0001**
Calcium (mg)	1014 (6.53)	1021 (25.4)	6.96 (26.6)	0.7941
Carbohydrate (gm)	257.5 (0.61)	261.9 (2.61)	4.41 (2.60)	0.0917
Cholesterol (mg)	218.5 (1.80)	229.5 (12.8)	10.97 (12.8)	0.3932
Choline (mg)	248.6 (1.47)	271.4 (10.4)	22.85 (10.4)	**0.0295**
Copper (mg)	0.98 (0.01)	1.09 (0.02)	0.10 (0.02)	**<0.0001**
Dietary fiber (gm)	13.55 (0.09)	15.39 (0.52)	1.84 (0.51)	**0.0005**
Energy (kcal)	1940 (9.70)	1963 (40.6)	23.89 (40.8)	0.5597
Folate, DFE (µg)	517.5 (4.72)	527.0 (19.4)	9.47 (19.2)	0.6229
Iron (mg)	14.10 (0.10)	14.46 (0.35)	0.36 (0.36)	0.3143
Lutein + zeaxanthin (mcg)	781.3 (19.0)	1036 (96.6)	255.2 (98.0)	**0.0103**
Lycopene (mcg)	4305 (91.9)	14,644 (854)	10,338 (874)	**<0.0001**
Magnesium (mg)	231.7 (1.10)	263.3 (5.41)	31.64 (5.44)	**<0.0001**
Niacin (mg)	21.24 (0.13)	21.72 (0.49)	0.49 (0.50)	0.3342
Phosphorus (mg)	1257 (4.86)	1279 (26.4)	22.07 (26.4)	0.4056
Potassium (mg)	2174 (10.7)	2393 (48.5)	218.9 (48.9)	**<0.0001**
Protein (gm)	68.44 (0.26)	70.85 (1.55)	2.42 (1.54)	0.1201
Riboflavin (Vitamin B2) (mg)	2.00 (0.01)	1.98 (0.04)	−0.03 (0.05)	0.5403
Sodium (mg)	3073 (13.7)	3042 (55.3)	−31.60 (57.1)	0.5809
Thiamin (Vitamin B1) (mg)	1.55 (0.01)	1.59 (0.04)	0.04 (0.04)	0.3126
Total Folate (mcg)	368.1 (2.95)	383.7 (12.0)	15.55 (12.0)	0.1964
Total fat (gm)	73.01 (0.25)	71.34 (0.85)	−1.67 (0.85)	0.0517
Total MUFA (gm)	25.58 (0.10)	24.68 (0.38)	−0.90 (0.38)	**0.0210**
Total PUFA (gm)	15.40 (0.09)	15.92 (0.49)	0.52 (0.50)	0.3000
Total SFA (gm)	25.58 (0.11)	24.20 (0.59)	−1.38 (0.59)	**0.0208**
Total sugars (gm)	124.7 (0.64)	127.3 (2.74)	2.55 (2.72)	0.3492
Vitamin A (RE)	585.9 (5.31)	673.9 (27.6)	88.06 (27.6)	**0.0018**
Vitamin B12 (mcg)	4.85 (0.04)	4.95 (0.25)	0.10 (0.26)	0.6925
Vitamin B6 (mg)	1.71 (0.01)	1.82 (0.06)	0.11 (0.06)	0.0524
Vitamin C (mg)	77.89 (1.11)	101.3 (4.64)	23.42 (4.51)	**<0.0001**
Vitamin D (D2 + D3) (µg)	5.56 (0.06)	5.51 (0.32)	−0.05 (0.33)	0.8874
Vitamin E (ATE) (mg)	6.64 (0.08)	7.37 (0.51)	0.73 (0.50)	0.1459
Vitamin K	61.84 (1.06)	72.17 (4.42)	10.34 (4.40)	**0.0204**
Zinc (mg)	10.25 (0.07)	10.32 (0.29)	0.06 (0.29)	0.8254

Data source: NHANES 2003–2018; subjects 2 to 18 years old with complete, reliable dietary recall on Day 1 (*n* = 21,004). ^a^ Values are adjusted for age, gender, ethnicity (Mexican American, other Hispanic, White, Black, Other), poverty income ratio, physical activity level (sedentary, moderate, vigorous), energy intake (except for energy). ^b^ *p* value for difference of intake between watermelon consumers and non-consumers; Bolded values are significantly different, *p* < 0.05; SE = standard error, MUFA = monounsaturated fatty acids, PUFA = polyunsaturated fatty acids, SFA = saturated fatty acids.

**Table 7:** Energy and nutrient intakes in adult watermelon consumers (*n* = 792) and non-consumers (*n* = 31,569), NHANES 2003–2018, gender combined data.

Nutrients ^a^	Non-Consumer (*n* = 31,569)	Consumer (*n* = 792)		
	Mean (SE)	Mean (SE)	Beta (SE)	*p* ^b^
Added sugars (tsp eq)	18.24 (0.16)	16.25 (0.65)	−1.99 (0.66)	**0.0030**
Beta-carotene (mcg)	2136 (42.8)	3681 (245)	1545 (247)	**<0.0001**
Beta-cryptoxanthin (mcg)	88.82 (1.96)	350.8 (22.6)	261.9 (23.0)	**<0.0001**
Calcium (mg)	960.1 (4.94)	948.1 (21.9)	−11.94 (22.3)	0.5930
Carbohydrate (gm)	257.3 (0.58)	270.5 (2.72)	13.22 (2.85)	**<0.0001**
Cholesterol (mg)	292.6 (1.91)	287.6 (9.46)	−5.01 (9.56)	0.6009
Choline (mg)	335.6 (1.38)	347.2 (6.42)	11.58 (6.48)	0.0768
Copper (mg)	1.30 (0.01)	1.36 (0.03)	0.06 (0.03)	**0.0365**
Dietary fiber (gm)	16.71 (0.12)	17.95 (0.35)	1.24 (0.37)	**0.0012**
Energy (kcal)	2160 (6.75)	2206 (35.5)	46.53 (36.2)	0.2014
Folate, DFE (µg)	534.5 (2.91)	559.6 (19.1)	25.15 (19.1)	0.1900
Iron (mg)	15.11 (0.06)	15.44 (0.33)	0.33 (0.33)	0.3159
Lutein + zeaxanthin (mcg)	1566 (39.1)	1940 (157)	373.4 (154)	**0.0171**
Lycopene (mcg)	5186 (89.8)	18,463 (1009)	13,278 (995)	**<0.0001**
Magnesium (mg)	301.9 (1.39)	323.7 (5.11)	21.78 (5.32)	**0.0001**
Niacin (mg)	25.87 (0.11)	25.82 (0.46)	−0.06 (0.47)	0.9004
Phosphorus (mg)	1385 (4.02)	1364 (16.2)	−20.21 (16.5)	0.2231
Potassium (mg)	2691 (10.2)	3005 (43.0)	314.0 (42.8)	**<0.0001**
Protein (gm)	83.17 (0.26)	82.95 (1.00)	−0.22 (1.00)	0.8232
Riboflavin (Vitamin B2) (mg)	2.20 (0.01)	2.16 (0.05)	−0.04 (0.05)	0.4039
Sodium (mg)	3599 (9.17)	3548 (67.7)	−50.37 (70.1)	0.4736
Thiamin (Vitamin B1) (mg)	1.64 (0.01)	1.76 (0.04)	0.13 (0.04)	**0.0037**
Total Folate (mcg)	405.0 (2.09)	427.8 (11.9)	22.84 (11.9)	0.0574
Total fat (gm)	83.50 (0.19)	78.85 (1.05)	−4.65 (1.09)	**<0.0001**
Total MUFA (gm)	29.91 (0.08)	28.18 (0.46)	−1.73 (0.47)	**0.0003**
Total PUFA (gm)	18.81 (0.09)	18.05 (0.38)	−0.77 (0.38)	**0.0478**
Total SFA (gm)	27.33 (0.09)	25.48 (0.51)	−1.85 (0.52)	**0.0005**
Total sugars (gm)	115.0 (0.53)	125.9 (3.21)	10.97 (3.30)	**0.0011**
Vitamin A (RE)	630.9 (6.56)	723.0 (29.6)	92.14 (30.4)	**0.0030**
Vitamin B12 (mcg)	5.21 (0.05)	4.70 (0.16)	−0.51 (0.16)	**0.0021**
Vitamin B6 (mg)	2.09 (0.01)	2.17 (0.06)	0.08 (0.06)	0.1886
Vitamin C (mg)	81.53 (0.97)	123.0 (4.65)	41.45 (4.76)	**<0.0001**
Vitamin D (D2 + D3) (µg)	4.63 (0.05)	4.34 (0.25)	−0.29 (0.25)	0.2541
Vitamin E (ATE) (mg)	8.47 (0.07)	8.31 (0.24)	−0.16 (0.24)	0.5236
Vitamin K	112.2 (1.95)	129.7 (9.27)	17.49 (9.00)	0.0544
Zinc (mg)	11.75 (0.06)	11.63 (0.27)	−0.12 (0.27)	0.6511

Data source: NHANES 2003–2018; subjects 19 years and older with complete, reliable dietary recall on Day 1 (*n* = 35,126). ^a^ Values are adjusted for age, gender, ethnicity (Mexican American, other Hispanic, White, Black, Other), poverty income ratio, physical activity level (sedentary, moderate, vigorous), smoking status, energy intake (except for energy). ^b^ *p* value for difference of intake between watermelon consumers and non-consumers; Bolded values are significantly different, *p* < 0.05; SE = standard error, MUFA = monounsaturated fatty acids, PUFA = polyunsaturated fatty acids, SFA = saturated fatty acids.

## Data Availability

The data presented in this study are from publicly available data in NHANES and other additional data are available in the article and supplementary material.

## Supplementary Materials

The following supporting information can be downloaded at: https://www.mdpi.com/article/10.3390/nu14224883/s1, Table S1: Watermelon intake grams per day per capita among children (2–18 years) and adults (19+ years), NHANES 2003–2018. Table S2: Association of watermelon consumption with Healthy Eating Index-2015 total and sub-component scores in children, NHANES 2003–2018, gender combined data. Table S3: Association of watermelon consumption with Healthy Eating Index-2015 total and sub-component score in adults, NHANES 2003–2018, gender combined data. Table S4: Energy and nutrient intakes in children watermelon consumers (*n* = 513) and non-consumers (*n* = 18,821), NHANES 2003–2018, gender combined data. Table S5: Energy and nutrient intakes in adult watermelon consumers (*n* = 792) and non-consumers (*n* = 31,569), NHANES 2003–2018, gender combined data.
