# Supplementary material for: Watermelon Intake Is Associated with Increased Nutrient Intake and Higher Diet Quality in Adults and Children, NHANES 2003–2018
Source: Nutrients. 2022 Nov 18;14(22):4883. doi: 10.3390/nu14224883 (PMC9692283; doi:10.3390/nu14224883)
Supplement: Supplementary file 1 [file nutrients-14-04883-s001.zip › nutrients-1974641-supplementary.pdf]

Supplementary Table S1. Watermelon intake grams per day per capita among children (2-18 years) and adults (19+ years), NHANES 2003-2018

	2-18 y			19+ y		
**NHANES Cycle**	**All (n=21,004)**	**Male (n=10,541)**	**Female (n=10,463)**	**All (n=35,126)**	**Male (n=17,226)**	**Female (n=17,900)**
**2003-2018**	7.51 (0.81)	8.27 (1.22)	6.72 (0.87)	7.29 (0.59)	6.96 (0.77)	7.61 (0.71)
**2003-2004**	4.34 (0.86)	3.90 (1.59)	4.81 (1.50)	7.07 (2.40)	7.70 (2.93)	6.47 (2.47)
**2005-2006**	6.32 (1.00)	4.97 (1.67)	7.75 (1.49)	4.44 (0.71)	4.18 (0.88)	4.69 (1.21)
**2007-2008**	7.57 (3.43)	6.43 (2.80)	8.71 (4.54)	6.22 (1.48)	7.73 (2.32)	4.89 (1.09)
**2009-2010**	7.45 (1.45)	7.87 (2.23)	7.04 (2.67)	8.01 (1.23)	6.67 (2.17)	9.28 (1.22)
**2011-2012**	8.78 (2.10)	11.1 (3.66)	6.34 (1.17)	5.73 (1.28)	5.17 (1.80)	6.27 (1.44)
**2013-2014**	7.40 (2.13)	10.1 (3.15)	4.60 (1.62)	8.31 (1.60)	6.88 (2.32)	9.67 (1.68)
**2015-2016**	7.88 (2.61)	10.2 (5.93)	5.73 (0.98)	9.36 (2.26)	6.23 (1.14)	12.4 (3.69)
**2017-2018**	10.2 (3.13)	11.6 (4.23)	8.71 (3.28)	8.77 (1.71)	10.8 (3.00)	6.80 (1.40)
	**Beta (SE)**	**Beta (SE)**	**Beta (SE)**	**Beta (SE)**	**Beta (SE)**	**Beta (SE)**
	0.59 (0.35)	1.12 (0.55)	0.04 (0.37)	0.49 (0.29)	0.35 (0.38)	0.62 (0.34)
**Cycle Trend**	**P[a]**	**P**	**P**	**P**	**P**	**P**
	0.0979	**0.0427**	0.9138	0.0962	0.3532	0.0683

Data presented as mean (standard error). [a]p value for difference between NHANES 2003-2004 and 2017-2018 cycles. Bolded values are significantly different, p<0.05; SE = standard error.

Supplementary Table S2. Association of watermelon consumption with Healthy Eating Index-2015 total and sub-component scores in children, NHANES 2003-2018, gender combined data

**Healthy Eating Index 2015 Components[a]**	**Non-Consumer (n=18,821) Mean (SE)**	**Consumer (n=513) Mean (SE)**	**Beta (SE)**	**P[b]**
Component 1 – total vegetables	2.15 (0.01)	2.14 (0.06)	-0.01 (0.06)	0.8883
Component 2 - greens and beans	0.87 (0.03)	1.01 (0.13)	0.14 (0.13)	0.2656
Component 3 - total fruit	2.46 (0.02)	4.18 (0.11)	1.71 (0.12)	**<0.0001**
Component 4 - whole fruit	2.17 (0.02)	4.47 (0.09)	2.30 (0.09)	**<0.0001**
Component 5 - whole grains	2.31 (0.05)	2.63 (0.18)	0.32 (0.18)	0.0863
Component 6 - dairy	6.89 (0.03)	6.83 (0.11)	-0.06 (0.11)	0.6092
Component 7 - total protein foods	3.56 (0.02)	3.70 (0.10)	0.14 (0.10)	0.1563
Component 8 - seafood and plant protein	1.58 (0.03)	1.89 (0.17)	0.31 (0.17)	0.0699
Component 9 - fatty acid ratio	3.83 (0.04)	4.01 (0.24)	0.18 (0.24)	0.4640
Component 10 - sodium	4.96 (0.05)	5.14 (0.21)	0.17 (0.21)	0.4089
Component 11 - refined grain	5.18 (0.05)	4.99 (0.29)	-0.19 (0.28)	0.5163
Component 12 - saturated fat	5.42 (0.04)	5.78 (0.22)	0.35 (0.22)	0.1120
Component 13 - added sugar	5.68 (0.04)	6.39 (0.18)	0.71 (0.18)	**0.0001**
HEI-2015 total score	47.07 (0.16)	53.17 (0.78)	6.10 (0.79)	**<0.0001**

Data source: NHANES 2003-2018; subjects 2 to 18 years old with complete, reliable dietary recall on Day 1 (N=21,004). [a]Values are adjusted for age, gender, ethnicity (Mexican American, other Hispanic, White, Black, Other), poverty income ratio, physical activity level (sedentary, moderate, vigorous), total

vegetables, non-watermelon fruit, and total dairy. [b]p value for difference of intake between watermelon consumers and non-consumers; Bolded values are significantly different, p<0.05; SE = standard error.

Supplementary Table S3. Association of watermelon consumption with Healthy Eating Index-2015 total and sub-component score in adults, NHANES 2003-2018, gender combined data

**Healthy Eating Index 2015 Components[a]**	**Non-Consumer (n=31,569) Mean (SE)**	**Consumer (n=792) Mean (SE)**	**Beta (SE)**	**P[b]**
Component 1 – total vegetables	3.08 (0.01)	3.12 (0.06)	0.05 (0.06)	0.4192
Component 2 - greens and beans	1.49 (0.02)	1.53 (0.12)	0.04 (0.12)	0.7403
Component 3 - total fruit	1.99 (0.01)	4.12 (0.09)	2.13 (0.09)	**<0.0001**
Component 4 - whole fruit	1.98 (0.02)	4.36 (0.08)	2.38 (0.08)	**<0.0001**
Component 5 - whole grains	2.40 (0.04)	2.40 (0.15)	0.004 (0.16)	0.9788
Component 6 - dairy	5.08 (0.02)	5.12 (0.12)	0.03 (0.12)	0.7772
Component 7 - total protein foods	4.21 (0.01)	4.18 (0.05)	-0.03 (0.05)	0.5822
Component 8 - seafood and plant protein	2.32 (0.02)	2.13 (0.14)	-0.18 (0.14)	0.1891
Component 9 - fatty acid ratio	4.96 (0.03)	5.13 (0.16)	0.17 (0.16)	0.2941
Component 10 - sodium	4.23 (0.03)	4.59 (0.19)	0.37 (0.19)	0.0541
Component 11 - refined grain	6.17 (0.03)	6.24 (0.18)	0.08 (0.19)	0.6948
Component 12 - saturated fat	5.87 (0.03)	6.49 (0.16)	0.62 (0.16)	**0.0003**
Component 13 - added sugar	6.55 (0.04)	6.95 (0.17)	0.41 (0.17)	**0.0187**
HEI-2015 total score	50.32 (0.14)	56.38 (0.58)	6.06 (0.60)	**<0.0001**

Data source: NHANES 2003-2018; subjects 19 years and older with complete, reliable dietary recall on Day 1 (N=35,126). [a]Values are adjusted for age, gender, ethnicity (Mexican American, other Hispanic, White, Black, Other), poverty income ratio, physical activity level (sedentary, moderate, vigorous), smoking status, total vegetables, non-watermelon fruit, and total dairy. [b]p value for difference of intake between watermelon consumers and non-consumers; Bolded values are significantly different, p<0.05; SE = standard error.

Supplementary Table S4**.** Energy and nutrient intakes in children watermelon consumers (n=513) and non-consumers (n=18,821), NHANES 2003-2018, gender combined data

**Nutrients[a]**	**Non-Consumer (n=18,821) Mean (SE)**	**Consumer (n=513) Mean (SE)**	**Beta (SE)**	**P[b]**
Added sugars (tsp eq)	18.90 (0.14)	16.27 (0.63)	-2.63 (0.63)	**0.0001**
Beta-carotene (mcg)	1,155 (26.5)	2,160 (167.8)	1,005 (169.0)	**<0.0001**
Beta-cryptoxanthin (mcg)	78.86 (2.56)	264.0 (17.3)	185.1 (17.2)	**<0.0001**
Calcium (mg)	1,013 (3.09)	1,059 (18.4)	45.60 (18.3)	**0.0141**
Carbohydrate (gm)	257.5 (0.58)	260.6 (2.34)	3.04 (2.34)	0.1962
Cholesterol (mg)	218.5 (1.81)	230.2 (12.7)	11.71 (12.7)	0.3582
Choline (mg)	248.6 (1.45)	271.4 (10.3)	22.78 (10.3)	**0.0290**
Copper (mg)	0.98 (0.01)	1.08 (0.02)	0.10 (0.02)	**<0.0001**
Dietary fiber (gm)	13.56 (0.08)	15.19 (0.42)	1.63 (0.41)	**0.0001**
Energy (kcal)	1,939 (8.49)	1,971 (35.2)	31.96 (35.7)	0.3722
Folate, DFE (μg)	517.4 (4.62)	531.9 (19.9)	14.50 (19.4)	0.4572
Iron (mg)	14.10 (0.10)	14.51 (0.35)	0.41 (0.36)	0.2515

Lutein + zeaxanthin (mcg)	782.2 (17.5)	1,009 (92.9)	226.5 (95.6)	**0.0194**
Lycopene (mcg)	4,307 (87.6)	14,590 (848)	10,283 (867)	**<0.0001**
Magnesium (mg)	231.7 (0.96)	263.3 (4.99)	31.65 (4.99)	**<0.0001**
Niacin (mg)	21.24 (0.13)	21.61 (0.50)	0.37 (0.51)	0.4631
Phosphorus (mg)	1,257 (3.72)	1,303 (20.1)	46.66 (19.9)	**0.0203**
Potassium (mg)	2,175 (7.24)	2,384 (39.1)	209.5 (39.0)	**<0.0001**
Protein (gm)	68.42 (0.26)	71.41 (1.44)	2.99 (1.43)	**0.0390**
Riboflavin (Vitamin B2) (mg)	2.00 (0.01)	2.02 (0.03)	0.02 (0.03)	0.5201
Sodium (mg)	3,073 (13.5)	3,045 (51.8)	-28.84 (53.5)	0.5905
Thiamin (Vitamin B1) (mg)	1.55 (0.01)	1.60 (0.04)	0.05 (0.04)	0.1758
Total Folate (mcg)	368.0 (2.87)	385.9 (12.1)	17.89 (12.0)	0.1370
Total fat (gm)	73.00 (0.22)	71.62 (0.78)	-1.38 (0.78)	0.0814
Total MUFA (gm)	25.58 (0.10)	24.68 (0.35)	-0.90 (0.35)	**0.0123**
Total PUFA (gm)	15.41 (0.08)	15.76 (0.46)	0.35 (0.46)	0.4470
Total SFA (gm)	25.56 (0.10)	24.63 (0.49)	-0.93 (0.49)	0.0604
Total sugars (gm)	124.8 (0.63)	126.6 (2.80)	1.89 (2.77)	0.4958
Vitamin A (RE)	585.4 (4.77)	687.1 (23.3)	101.7 (23.3)	**<0.0001**
Vitamin B12 (mcg)	4.84 (0.04)	5.08 (0.24)	0.24 (0.24)	0.3264
Vitamin B6 (mg)	1.71 (0.01)	1.81 (0.05)	0.10 (0.05)	0.0611
Vitamin C (mg)	78.03 (0.79)	96.93 (4.10)	18.90 (4.15)	**<0.0001**
Vitamin D (D2+D3) (µg)	5.55 (0.04)	5.79 (0.27)	0.24 (0.27)	0.3735
Vitamin E (ATE) (mg)	6.65 (0.08)	7.27 (0.48)	0.62 (0.47)	0.1882
Vitamin K	61.89 (0.97)	70.45 (3.61)	8.56 (3.62)	**0.0197**
Zinc (mg)	10.25 (0.07)	10.44 (0.27)	0.19 (0.28)	0.4920

Data source: NHANES 2003-2018; subjects 2 to 18 years old with complete, reliable dietary recall on Day 1 (N=21,004). [a]Values are adjusted for age, gender, ethnicity (Mexican American, other Hispanic, White, Black, Other), poverty income ratio, physical activity level (sedentary, moderate, vigorous), energy intake (except for energy), total vegetables, non-watermelon fruit, and total dairy. [b]p value for difference of intake between watermelon consumers and non-consumers; Bolded values are significantly different, p<0.05; SE = standard error, MUFA = monounsaturated fatty acids, PUFA = polyunsaturated fatty acids, SFA = saturated fatty acids.

Supplementary Table S5. Energy and nutrient intakes in adult watermelon consumers (n=792) and non-consumers (n=31,569), NHANES 2003-2018, gender combined data

**Nutrients[a]**	**Non-Consumer (n=31,569) Mean (SE)**	**Consumer (n=792) Mean (SE)**	**Beta (SE)**	**P[b]**
Added sugars (tsp eq)	18.22 (0.13)	16.92 (0.62)	-1.30 (0.64)	**0.0435**
Beta-carotene (mcg)	2,147 (34.6)	3,236 (227)	1,089 (231)	**<0.0001**
Beta-cryptoxanthin (mcg)	89.15 (1.73)	337.6 (23.0)	248.5 (23.3)	**<0.0001**
Calcium (mg)	959.6 (2.41)	965.7 (11.4)	6.02 (11.4)	0.5999
Carbohydrate (gm)	257.3 (0.57)	269.2 (2.28)	11.88 (2.40)	**<0.0001**
Cholesterol (mg)	292.6 (1.89)	289.7 (9.25)	-2.84 (9.32)	0.7607
Choline (mg)	335.8 (1.38)	342.1 (6.55)	6.27 (6.71)	0.3519

Copper (mg)	1.30 (0.01)	1.31 (0.02)	0.01 (0.03)	0.6286
Dietary fiber (gm)	16.73 (0.08)	16.92 (0.31)	0.19 (0.32)	0.5512
Energy (kcal)	2,161 (6.34)	2,157 (29.3)	-3.67 (30.3)	0.9036
Folate, DFE (μg)	534.6 (2.81)	554.4 (18.8)	19.79 (18.9)	0.2961
Iron (mg)	15.11 (0.05)	15.33 (0.33)	0.22 (0.34)	0.5163
Lutein + zeaxanthin (mcg)	1,574 (33.1)	1,617 (158)	42.73 (158)	0.7877
Lycopene (mcg)	5,197 (84.1)	18,024 (971)	12,827 (960)	**<0.0001**
Magnesium (mg)	302.1 (1.10)	315.3 (4.77)	13.16 (4.95)	**0.0089**
Niacin (mg)	25.88 (0.11)	25.64 (0.45)	-0.24 (0.45)	0.6031
Phosphorus (mg)	1,384 (3.16)	1,371 (13.0)	-13.65 (13.5)	0.3138
Potassium (mg)	2,694 (5.69)	2,878 (35.1)	184.0 (35.8)	**<0.0001**
Protein (gm)	83.17 (0.25)	82.80 (1.01)	-0.38 (1.02)	0.7129
Riboflavin (Vitamin B2) (mg)	2.20 (0.01)	2.18 (0.05)	-0.02 (0.05)	0.6549
Sodium (mg)	3,600 (8.82)	3,509 (63.2)	-90.85 (64.8)	0.1632
Thiamin (Vitamin B1) (mg)	1.64 (0.01)	1.75 (0.04)	0.12 (0.04)	**0.0070**
Total Folate (mcg)	405.2 (1.92)	419.1 (11.8)	13.89 (11.9)	0.2464
Total fat (gm)	83.49 (0.19)	79.24 (0.91)	-4.24 (0.94)	**<0.0001**
Total MUFA (gm)	29.91 (0.08)	28.24 (0.42)	-1.66 (0.43)	**0.0002**
Total PUFA (gm)	18.82 (0.09)	17.79 (0.34)	-1.03 (0.35)	**0.0040**
Total SFA (gm)	27.31 (0.08)	26.04 (0.43)	-1.27 (0.44)	**0.0046**
Total sugars (gm)	115.0 (0.54)	126.1 (2.83)	11.11 (2.91)	**0.0002**
Vitamin A (RE)	631.6 (5.94)	696.6 (27.2)	65.08 (28.1)	**0.0220**
Vitamin B12 (mcg)	5.21 (0.05)	4.81 (0.16)	-0.40 (0.16)	**0.0144**
Vitamin B6 (mg)	2.09 (0.01)	2.11 (0.06)	0.01 (0.05)	0.8311
Vitamin C (mg)	81.83 (0.62)	111.1 (3.83)	29.27 (3.96)	**<0.0001**
Vitamin D (D2+D3) (μg)	4.62 (0.05)	4.47 (0.25)	-0.15 (0.25)	0.5394
Vitamin E (ATE) (mg)	8.47 (0.06)	7.97 (0.22)	-0.50 (0.23)	**0.0309**
Vitamin K	112.7 (1.64)	110.5 (9.23)	-2.17 (9.23)	0.8141
Zinc (mg)3	11.75 (0.06)	11.60 (0.26)	-0.15 (0.26)	0.5613

Data source: NHANES 2003-2018; subjects 19 years and older with complete, reliable dietary recall on Day 1 (N=35,126). [a]Values are adjusted for age, gender, ethnicity (Mexican American, other Hispanic, White, Black, Other), poverty income ratio, physical activity level (sedentary, moderate, vigorous), smoking status, energy intake (except for energy), total vegetables, non-watermelon fruit, and total dairy. [b]p value for difference of intake between watermelon consumers and non-consumers; Bolded values are significantly different, $p<0.05$; SE = standard error, MUFA = monounsaturated fatty acids, PUFA = polyunsaturated fatty acids, SFA = saturated fatty acids.
